# Rare Earth Fluorescent Composite Hydrogel with Controllable Color Photoluminescence for Information Encryption

**DOI:** 10.3390/polym17111534

**Published:** 2025-05-30

**Authors:** Jiajia Du, Daohai Zhang, Teng Zhou, Kunlan Diao, Zhi Lei

**Affiliations:** School of Chemical Engineering, Guizhou Minzu University, Guiyang 550025, China; jiajiadu2023@163.com (J.D.); 18886044278@163.com (T.Z.); 17608592049@163.com (K.D.); 18850330437@163.com (Z.L.)

**Keywords:** hydrogel, rare earth, color control, alkaline pH response, information encryption

## Abstract

In the context of the information age, the need for data security and confidentiality is becoming increasingly urgent. In this study, polyvinyl alcohol (PVA) and polyethylene glycol (PEG) were used as the matrix, and a PVA/PEG/rare earth composite hydrogel material with controllable photoluminescence color was successfully developed by incorporating rare earth ion doping. Through scanning electron microscopy (SEM), X-ray photoelectronic spectroscopy (XPS), X-ray diffraction (XRD), and fluorescence spectroscopy, it was confirmed that the introduction of lanthanide metal light-emitting units makes the material’s photoluminescence color adjustable from red to green, significantly improves the mechanical properties, and the compressive strength is increased from 17.6 MPa to 23 MPa, representing a 30.7% improvement. In addition, the material exhibits excellent alkaline pH response characteristics; as the concentration of NaOH solution increases, the luminous intensity gradually decays to complete quenching. Based on the adjustable light color and dynamic response characteristics, the material can realize information concealment and encryption through programmable light color changes, providing a new functional material solution for intelligent anti-counterfeiting and optical encryption.

## 1. Introduction

Today, with the rapid development of the information society, information security and privacy protection have become the focus of attention in various fields. Information encryption, as an important means of information protection, is of great significance in preventing information leakage and maintaining national security. Photoluminescent materials are widely used in fields such as anti-counterfeiting, information storage, and encryption due to their unique luminescent properties [1,2,3,4,5]. Among rare earth elements (Ln), Eu^3+^ and Tb^3+^ have significant advantages in photoluminescent materials due to their unique 4f electron transition characteristics: (1) High color purity: Eu^3+^’s ^5^D_0_→^7^F_2_ transition (615 nm) and Tb^3+^’s ^5^D_4_→^7^F_5_ transition (545 nm) correspond to high purity red and green light emissions, respectively, covering the core spectral region of the visible light range [6,7]. (2) Long fluorescence lifetime and high quantum efficiency: Compared with other Ln^3+^ (such as Ce^3+^ and Sm^3+^), Eu^3+^ and Tb^3+^ have larger excited state energy level spacing and a lower non-radiative transition probability, so they have a longer fluorescence lifetime (microseconds) and higher luminescence efficiency [8]. (3) Environmental stability: Their coordination structure is less sensitive to water molecules and oxygen, making them suitable for use in hydrogel systems. In contrast, the emission spectra of other Ln^3+^ (such as Dy^3+^ and Pr^3+^) often have multiple peaks overlapping or are located in the near-infrared region, which is not conducive to multi-color regulation and visualization applications [9].

Hydrogels are composed of hydrophilic chains that form a three-dimensional network structure through chemical cross-linking (such as covalent bonds and ionic bonds) or physical cross-linking (such as strong entanglements, microcrystals, and hydrogen bonds). These cross-linking points give the hydrogel excellent hydrophilicity and water retention properties [10,11,12]. Hydrogels have the characteristics of high purity, good biocompatibility, high mechanical strength, and simple preparation, and are widely used in biomedicine, environmental protection, and other fields [13,14,15,16,17]. Among them, polyvinyl alcohol (PVA) and polyethylene glycol (PEG) have good processability and stability and are ideal matrices for preparing hydrogel composites [18,19,20,21]. In recent years, hydrogels have attracted extensive attention as carriers of photoluminescent materials. Wang et al. [22] embedded rare earth nanoparticles into cellulose hydrogels to achieve multicolor fluorescence regulation; Zhao et al. [23] developed a rare earth-doped fluorescent film based on PVA for anti-counterfeiting labels. However, existing research still has the following limitations: the mechanical strength of traditional hydrogels is insufficient; the fluorescence color adjustment range is limited (usually relying on a single rare earth ion or static ratio); and the dynamic response characteristics are lacking. Therefore, it is still challenging to develop new fluorescent hydrogels with high mechanical properties, wide color gamut regulation, and dynamic response capabilities.

This study aims to prepare PVA/PEG/Ln composite hydrogel materials with controllable color photoluminescence properties and explore their applications in information encryption. The above limitations were overcome through the following innovations: (1) Synergistically enhanced mechanical properties: Through the coordination and cross-linking of Ln^3+^ and PVA/PEG chains, the compressive strength of the composite hydrogel was increased by 30.7% (from 17.6 MPa to 23 MPa). (2) Wide color gamut and dynamic regulation: The ligand 2,6-pyridinedicarboxylic acid (L) is used to chelate with Ln to form a luminescent complex Ln-L_3_, thereby improving the luminescence efficiency of Ln. L absorbs ultraviolet light energy through the antenna effect and is excited to the singlet excited state (S_1_), and then generates a long-lived triplet excited state (T_1_) through spin-forbidden intersystem crossing (ISC); the triplet state energy is efficiently transferred to the ff transition energy level of the lanthanide ions (Eu^3+^, Tb^3+^) through FRET (Figure S5) [24,25]. By adjusting the Eu^3+^/Tb^3+^ ratio (10:0 to 0:10), red–green continuously tunable multi-color luminescence was achieved, and the alkaline environment (NaOH) could dynamically regulate the fluorescence intensity until quenching. (3) Multifunctional information encryption application: Combining programmable light color changes with dynamic responses, a dual encryption strategy based on fluorescence sequences and alkaline trigger signals was realized, providing new ideas for high-security optical encryption materials.

## 2. Materials and Methods

### 2.1. Material Preparation

Polyvinyl alcohol (PVA, AR), polyethylene glycol-400 (PEG-400, AR), and 2,6-pyridinedicarboxylic acid (L, AR) were purchased from Aladdin Reagent (Shanghai, China), and terbium (III) trichloride hexahydrate (TbCl_3_·6H_2_O, 99.9%) and europium (III) nitrate hexahydrate (Eu (NO_3_)_3_·6H_2_O, 99.9%) were purchased from Beijing Huawei Ruike Co., Ltd. (Beijing, China). All purified water used in the study was prepared using water purifiers.

### 2.2. Preparation of PVA/PEG/Ln Hydrogel

PVA/PEG/Ln hydrogel was prepared via a simple hydrothermal method. First, prepare the PVA/PEG matrix according to the following steps: add 5 g PVA and 40 mL H_2_O in a round-bottomed flask, heat and stir in an oil bath at 90 °C for 2 h, add PEG-400 aqueous solution after cooling, and ensure that the PVA/PEG mass ratio is 5:5, stir for 0.5 h, and then pour into the mold for circulation freezing.

To prepare PVA/PEG/Ln hydrogel, 1 g of lanthanide salt and 4 g of ligand L were uniformly mixed into the PVA/PEG matrix solution. The mass ratios of Eu^3+^ and Tb^3+^ are 0:10, 2:8, 5:5, 8:2, and 10:0 to obtain hydrogels of different colors. After cyclic freezing, the PVA/PEG/Ln composite hydrogel material is obtained by washing with deionized water.

### 2.3. Characterization

The PVA/PEG/Ln composite hydrogel material was cut into small pieces and dried to prepare samples, and the samples were coated with Au powder using SEM (Quanta FEG 250, FEI Company, Hillsboro, OR, USA) for SEM observation. The FT-IR spectrometer (Thermo, Fisher Scientific, Waltham, MA, USA) was used to scan in the range of 600–3600 cm^−1^ to obtain the Fourier transform infrared spectrum. X-ray diffraction (XRD) (Rigaku Ultima IV, Tokyo, Japan) was performed using a diffractometer equipped with a Cu-Kα radiation source, with the data collection range of 5–90° and a scanning rate of 10°/min. X-ray photoelectron spectroscopy (XPS) was performed on a Thermo Scientific K-Alpha using Al Kα X-rays. We collected emission spectra of the samples over the range of 300–730 nm using a Hitachi (Tokyo, Japan) F-4600 fluorescence spectrophotometer at an excitation wavelength of 254 nm [26]. Mechanical tests were performed on cylindrical PVA/PEG/LN composite hydrogel samples using a general mechanical test bench (CMT 6104, Systems (China) Co., Shenzhen, China) at a load speed of 100 mm/min and a load speed of greater than 0.01. N Measurement starts the test machine to compress the sample to a positioning shift [27]. Mechanical tests were performed on cylindrical PVA/PEG/LN composite hydrogel samples using a general mechanical test bench (CMT 6104, Systems (Suzhou, China) Co.) at a load speed of 100 mm/min. The mechanical properties of the hydrogels were quantified using compressive stress (*λ*) and strain (*ε*):

(1) $$\lambda =-\frac{p}{A}*100\%$$

(2) $$\varepsilon =\frac{∆L}{L_{0}}*100\%=\frac{L_{0}-L_{t}}{L_{O}}$$

The moisture content (*W*) test uses and records the weight of the small piece of composite hydrogel material (*W*_1_) and the weight after drying (*W*_2_) according to the formula [28,29]:

(3) $$W=\frac{W_{1}-W_{2}}{W_{1}}*100\%$$

For the swelling rate test, the dried samples after the water content test are directly soaked in deionized water, and the weight of each sample is recorded at 1 h, 2 h, 3 h, 6 h, 12 h, and 24 h.

## 3. Results and Discussion

### 3.1. Structure and Morphology of PVA/PEG/Ln Composite Hydrogel Material

**Figure 1:**
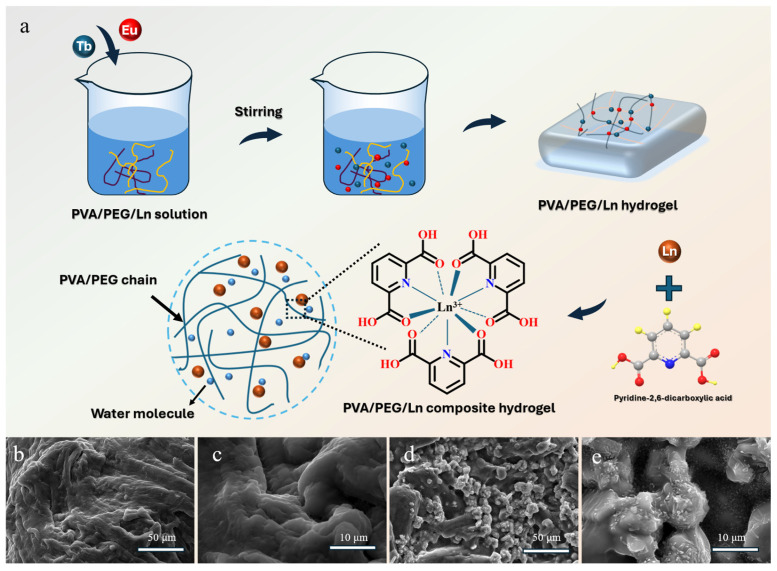
Schematic diagram of the preparation route of PVA/PEG/Ln composite hydrogel material (**a**); SEM images of PVA/PEG hydrogel (**b**,**c**); SEM image of PVA/PEG/Ln composite hydrogel material (Eu/Tb = 10:0) (**d**,**e**).

In this study, a color-controllable PVA/PEG/Ln composite hydrogel material was constructed using PVA and PEG-400 as matrices through a hydrothermal reaction (Figure 1a). SEM characterization revealed that the pure PVA/PEG hydrogel exhibited a continuous, uniform, and smooth surface morphology without cracks or particle protrusions (Figure 1b,c), where the fibrous network was stabilized by hydrogen bonds and chemical cross-linking between PVA and PEG chains (Figure 1c). Upon the introduction of Ln^3+^, the composite hydrogel displayed a distinct granular structure (Figure 1d), attributed to the interaction between Ln^3+^ and the PVA/PEG chains. Specifically, Ln^3+^ coordinated with the polymer chains via bonding interactions, refining the metal matrix grains and optimizing the interfacial properties. Simultaneously, the “antenna effect” [26,30,31] enabled efficient energy absorption and transfer to the lanthanide ion luminescent centers, enhancing both emission intensity and biocompatibility. This structural modulation not only endowed the hydrogel with programmable luminescence under specific light sources, but also reinforced its mechanical stability through the cross-linked network, offering a novel strategy for the synergistic optimization of structure and performance in light-responsive innovative materials.

### 3.2. Characterization of PVA/PEG/Ln Composite Hydrogel Material

**Figure 2:**
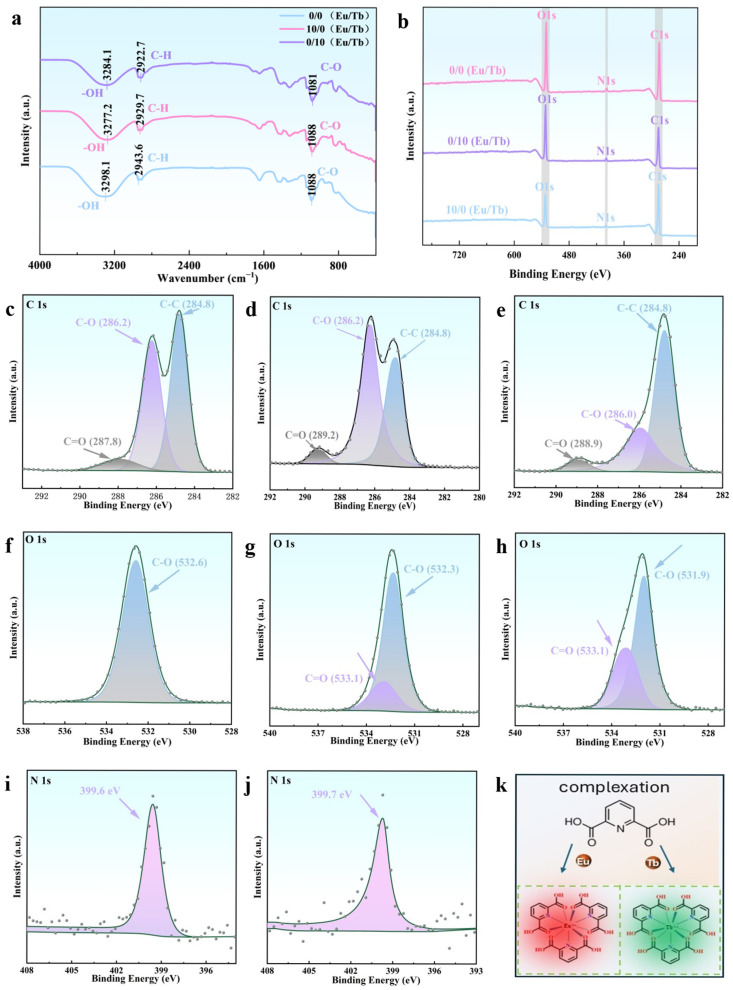
Dried hydrogel samples (0/0 Eu/Tb; 10/0 Eu/Tb; 0/10 Eu/Tb): FT-IR spectrum (**a**) and XPS scan (**b**); C1s spectra of XPS of PVA/PEG/Ln composite hydrogel materials: 0/0 Eu/Tb (**c**); 0/10 Eu/Tb (**d**); 10/0 Eu/Tb (**e**); O1s of XPS of PVA/PEG/Ln composite hydrogel material: 0/0 Eu/Tb (**f**); 0/10 Eu/Tb (**g**); 10/0 Eu/Tb (**h**); N1s spectra of XPS of PVA/PEG/Ln composite hydrogel material: 0/10 Eu/Tb (**i**); 10/0 Eu/Tb (**j**); and coordination diagram of Ln-L_3_ (**k**).

The FT-IR spectra of the PVA/PEG/Ln composite hydrogel materials exhibit high similarity (Figure 2a), indicating the minimal influence of Eu and Tb doping on infrared light absorption or scattering (Figure S1). For the pure PVA/PEG hydrogel, characteristic peaks at 3298 cm^−1^, 2944 cm^−1^, and 1088 cm^−1^ correspond to the stretching vibrations of the -OH, C-H, and C-O groups, respectively [32]. Upon introducing Ln-L_3_, a blue shift in the -OH stretching vibration is observed, which is due to the enhanced hydrogen bonding interaction between the hydroxyl group and the Ln^3+^ through the coordination effect [33,34]. The C-O and C-H vibration peaks further confirm the integrity of the PVA/PEG covalent backbone and demonstrate the compatibility of rare earth elements with the polymer matrix.

XPS analysis revealed the chemical composition and bonding characteristics of PVA/PEG/Ln composite hydrogels. Figure 2b and Figure S3 show that the main peak positions of the three groups of materials are consistent, indicating that the introduction of Ln-L3 did not significantly change the overall chemical environment of the polymer chain, but that its local interactions could be resolved by fine spectra. In the C1s spectrum (Figure 2c–e), the characteristic peaks at 284.8 eV (C-C), 286.2 eV (C-O), and 287.8 eV (C=O) confirm the structural characteristics of the PVA/PEG chain. It is worth noting that the introduction of Ln-L_3_ shifts the C=O peak to 288 eV (Figure S2), which is attributed to the hydrogen bonding or coordination between −COOH in Ln-L_3_ and the PVA/PEG chain, resulting in a change in the carbonyl electron cloud density [35,36]. For the O1s spectrum in Figure 2f–h, the 532.6 eV and 532.7 eV bands are attributed to the C-O and C=O peaks, respectively [23,35,37]. In the PVA/PEG/Ln hydrogel system, the C-O peak bands come from the alcoholic hydroxyl groups and ether bonds in the PVA/PEG chain, as well as possible ester bonds. C=O originates from trace oxidation products in the PVA/PEG chain and the carboxyl groups of Ln-L_3_. Since pure PVA/PEG has no nitrogen signal, the peak at 399.6 eV of the composite in the N1s spectrum (Figure 2i,j) is due to the pyridinic nitrogen in the Ln^3+^ complex, confirming that Ln-L_3_ is integrated into the polymer network via metal coordination. The process of combining Ln-L_3_ is shown in Figure 2k.

**Figure 3:**
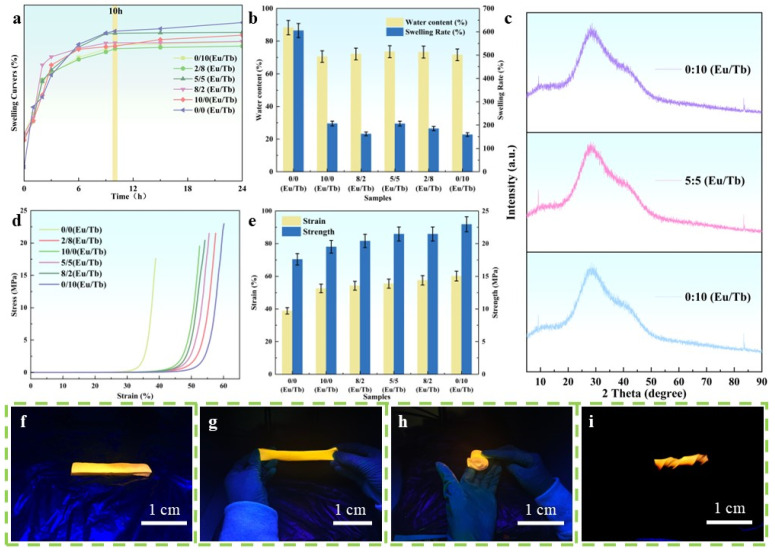
Performance characterization of polyvinyl alcohol/polyethylene glycol/rare earth composite hydrogel material: swelling ratio test graph (**a**); moisture content test graph (**b**); XRD spectrum of dried hydrogel samples (0/0 Eu/Tb; 10/0 Eu/Tb; 0/10 Eu/Tb) (**c**); strain and stress test graphs (**d**,**e**); toughness performance test of PVA/PEG/Ln composite hydrogel material: original diagram (**f**); stretch diagram (**g**); curl diagram (**h**); and twist diagram (**i**).

The difference in the diffraction peak position and intensity shown in the XRD pattern of the PVA/PEG/Ln composite hydrogel material reflects the difference in their internal structure and composition. As shown in Figure S4, the PVA/PEG hydrogel has obvious diffraction peaks at 16.58°, 22.48°, and 36.82°, indicating that the molecular chains form a three-dimensional network structure through orderly arrangement or aggregation. As can be seen from Figure 3c, Eu-L_3_ and Tb-L_3_ are isostructural.

The water content and swelling behavior of the hydrogel revealed the regulatory effect of Ln-L_3_ on the PVA/PEG network structure. As shown in Figure 3b, the water content of the PVA/PEG hydrogel was the highest (88.26%) when no rare earth was added, while the water content of the composite hydrogel was significantly reduced after the introduction of Ln-L_3_ (regardless of the Eu/Tb ratio), indicating that Ln^3+^ and the PVA/PEG chain formed a cross-linked network through coordination or hydrogen bonding, and that its steric hindrance effect inhibited water penetration. The swelling experiment (Figure 3a) further confirmed this mechanism: pure PVA/PEG showed a high swelling rate due to loose chain segments, while the introduction of Ln-L_3_ formed a dense and stable three-dimensional network through the synergistic cross-linking of carboxyl and pyridine groups, which significantly restricted the expansion of the chain segments and led to a decrease in the swelling rate. It is worth noting that the swelling curves of the composite hydrogels with different Eu/Tb ratios were slightly different, and all tended to stabilize after 10 h, indicating that the type and ratio of rare earth had limited effects on the cross-linking strength, and the coordination cross-linking structure dominated by Ln-L_3_ was universal [26].

Figure 3d,e are mechanical test diagrams of a PVA/PEG/Ln composite hydrogel material. The introduction of Ln-L_3_ improved the mechanical properties of the hydrogels, and with the increase in the proportion of Tb^3+^, the mechanical properties of the PVA/PEG/Ln (0/10 Eu/Tb) composite hydrogels reached their best, with a stress of up to 23 MPa, while that of PVA/PEG hydrogels only reached 17.6 MPa, which is 130.7% higher than that of the PVA/PEG hydrogel. This is attributed to the difference in the ionic radius and coordination number between Eu^3+^ and Tb^3+^ [26].

The prepared PVA/PEG/Ln composite hydrogel material has good toughness, as shown in Figure 3f–i. After a series of stretches and twists, it can finally return to its original state. This toughness mainly comes from two polymers of PVA and PEG. The introduction of Ln-L_3_ is effectively combined with the polymer matrix to form a more stable network structure. This enhanced connectivity helps composite hydrogel materials to better retain their shape and structural integrity when subjected to external forces, avoiding cracks or breakage. In addition, Ln elements may exist in the form of nanoparticles, which can fill the voids in the polymer matrix and act as a bridge, making the connection between the matrix tighter and more stable, showing higher toughness.

### 3.3. Photoluminescence Properties of PVA/PEG/Ln Composite Hydrogel Materials

**Figure 4:**
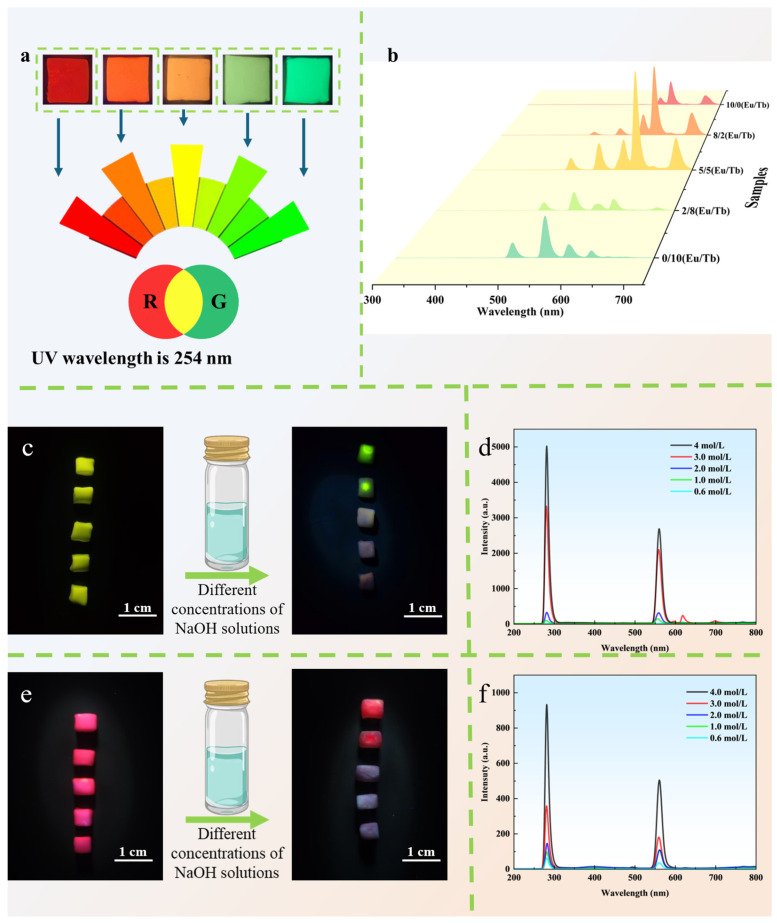
Luminescence images of PVA/PEG/Ln composite hydrogel materials with different ratios of Eu/Tb (10:0, 8:2, 5:5, 2:8, 0:10) under ultraviolet light (**a**); fluorescence spectra of PVA/PEG/Ln composite hydrogel materials at an excitation wavelength of 254 nm (**b**); 0/10 Eu/Tb composite hydrogel: color comparison before and after immersion in different concentrations of NaOH under UV light (**c**), fluorescence spectrum after immersion (**d**); 10/0 Eu/Tb composite hydrogel: color comparison before and after immersion in different concentrations of NaOH under UV light (**e**), and fluorescence spectrum after immersion (**f**).

The photoluminescent properties of the PVA/PEG/Ln composite hydrogels enable programmable multicolor emission by controlling the proportions of Eu^3+^ and Tb^3+^ (Figure 4a). When the Eu/Tb ratio varies from 10:0 to 0:10, the hydrogels exhibit red, yellow, orange, cyan, and green colors under 254 nm UV excitation. This phenomenon arises from the characteristic transitions of Eu^3+^ (^5^D_0_→^7^F_j_, j = 0–4, with emission peaks at 595, 620, and 698 nm) [1,38] and Tb^3+^ (^5^D_4_→^7^F_j_, j = 6–3, with emission peaks at 495, 548, 588, and 625 nm) (Figure 4b). As the proportion of Eu increases, the emission intensity of Tb^3+^ gradually decreases, while that of Eu^3+^ intensifies, suggesting competitive coordination or energy transfer effects between the two lanthanide ions. This confirms that the photoluminescent behavior can be precisely tuned by adjusting the type and concentration of Ln^3+^ [39].

Further studies reveal that luminescent properties are highly sensitive to external chemical environments. After immersing the hydrogels (Eu/Tb = 10:0 and 0:10) in NaOH solutions (0.4–4 mol/L), both the luminescence intensity and color diminish progressively with increasing alkali concentration (Figure 4c–f). This is attributed to OH− disrupting the coordination cross-linking points between Ln^3+^ and polymer chains (e.g., carboxyl and pyridyl groups), leading to network dissociation (evidenced by hydrogel surface collapse). Additionally, OH^−^ may perturb the electronic structure of the luminescent centers (e.g., Eu^3+^/Tb^3+^ coordination complexes) or increase coordinated water molecules, resulting in fluorescence quenching [40]. This pH-responsive behavior demonstrates that the hydrogel’s emission can be dynamically controlled by the alkali concentration, offering a novel strategy for designing advanced information encryption systems. By leveraging alkali-triggered reversible/irreversible fluorescence attenuation, multi-level anti-counterfeiting or time-dependent information storage modes can be achieved, highlighting the potential of these hydrogels in high-security photo functional applications.

### 3.4. The Application of Information Encryption

**Figure 5:**
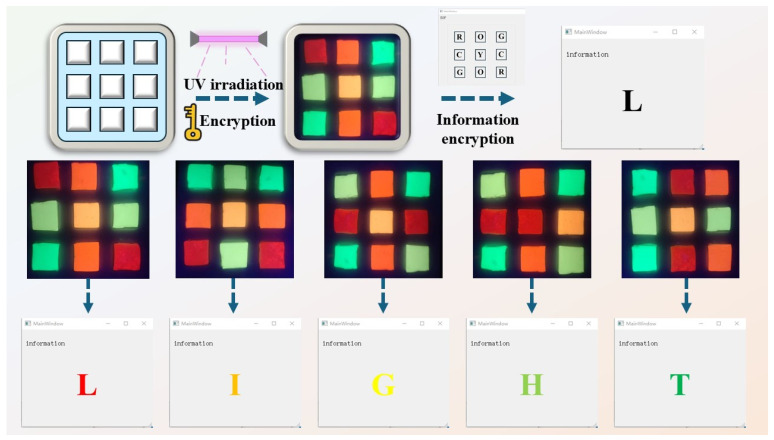
Schematic diagram of information encryption realized using PVA/PEG/Ln composite hydrogel materials with adjustable luminescence color.

During the encryption process, the fluorescence characteristics of the PVA/PEG/Ln composite hydrogel material were used to adjust the doping ratio of Eu and Tb (Eu/Tb = 10:0, 8:2, 5:5, 2:8, 0:10) when a high-purity fluorescence display of red (R), orange (O), yellow (Y), cyan (C), and green (G) was achieved. By placing these different colors of hydrogels on the information encryption board in a specific arrangement, we can encode letters into color sequences. The decryption process relies on specific wavelength sources (such as ultraviolet light) to excite the fluorescence of the hydrogel’s characteristics. Under natural light, the hydrogel does not show color. Use a light source at a specific wavelength to illuminate the information encryption plate and read the fluorescent color sequence of the hydrogel. Decode the color sequence into the corresponding letter or symbol through the color–letter correspondence preset by the custom Python script (developed during January-March 2025). For example, when the color sequence “ROGCYCGOR” is detected, the system outputs the letter “L”. As shown in Figure 5, the information “L” is output using the Python input color sequence “ROGCYCGOR” through the principle from left to right and top to bottom. Similarly, it inputs “GCGOYORCR”, “COGRYRGOC”, “COGRRYGOC”, and “GROCYCGRO” and outputs “I”, “G”, “H”, and “T”, and finally get “light”.

## 4. Conclusions

In this study, a PVA/PEG/Ln composite hydrogel with tunable fluorescence was synthesized via a simple hydrothermal method. By adjusting the concentration ratio of Eu^3+^ and Tb^3+^, a continuous fluorescence color transition from red to green was achieved, accompanied by enhanced mechanical properties (30.7% improvement in strength) and structural stability. The incorporation of Ln-L_3_ significantly increased the cross-linking density, suppressing swelling behavior and lowering the water content while reinforcing interfacial interactions through lanthanide ion coordination networks. Furthermore, the unique fluorescence response of the material enables information encryption systems; multicolor sequences encoded by Eu/Tb ratios effectively conceal data, and alkali-triggered fluorescence attenuation allows for the dynamic control of optical signals. These findings highlight the potential of PVA/PEG/Ln composite hydrogels as intelligent materials for high-security information storage and anti-counterfeiting technologies.

## Data Availability

The data used to support the findings of this study are included within the article.

## Supplementary Materials

The following supporting information can be downloaded at: https://www.mdpi.com/article/10.3390/polym17111534/s1, Figure S1. FT-IR spectrum of Ln-L3 (10/0 Eu/Tb; 5/5 Eu/Tb; 0/10 Eu/Tb). Figure S2. XPS C 1s, N 1s, and O 1s fitting results of 0/10 Eu/Tb (a); 5/5 Eu/Tb (b); 10/0 Eu/Tb (c). Figure S3. XPS scan of Ln-L3 (10/0 Eu/Tb; 5/5 Eu/Tb; 0/10 Eu/Tb). Figure S4. XRD patterns of PVA/PEG hydrogel. Figure S5. The energy transfer diagram.
