# Supplementary material for: Rare Earth Fluorescent Composite Hydrogel with Controllable Color Photoluminescence for Information Encryption
Source: Polymers (Basel). 2025 May 30;17(11):1534. doi: 10.3390/polym17111534 (PMC12157208; doi:10.3390/polym17111534)
Supplement: Supplementary file 1 [file polymers-17-01534-s001.zip › polymers-3628168-supplementary.pdf]

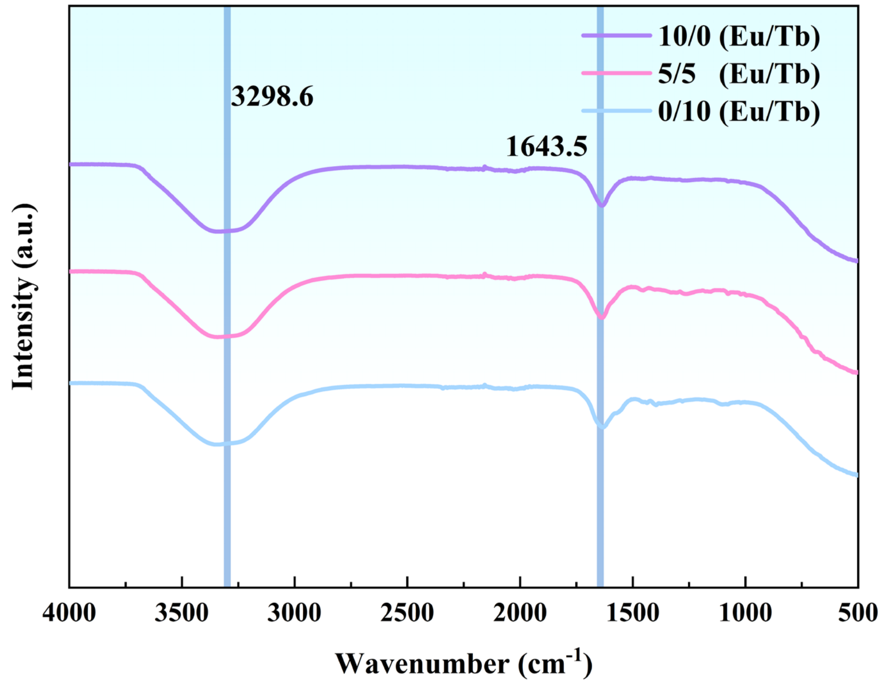


**Figure S1.** FT-IR spectrum of Ln-L3 (10/0 Eu/Tb; 5/5 Eu/Tb; 0/10 Eu/Tb).

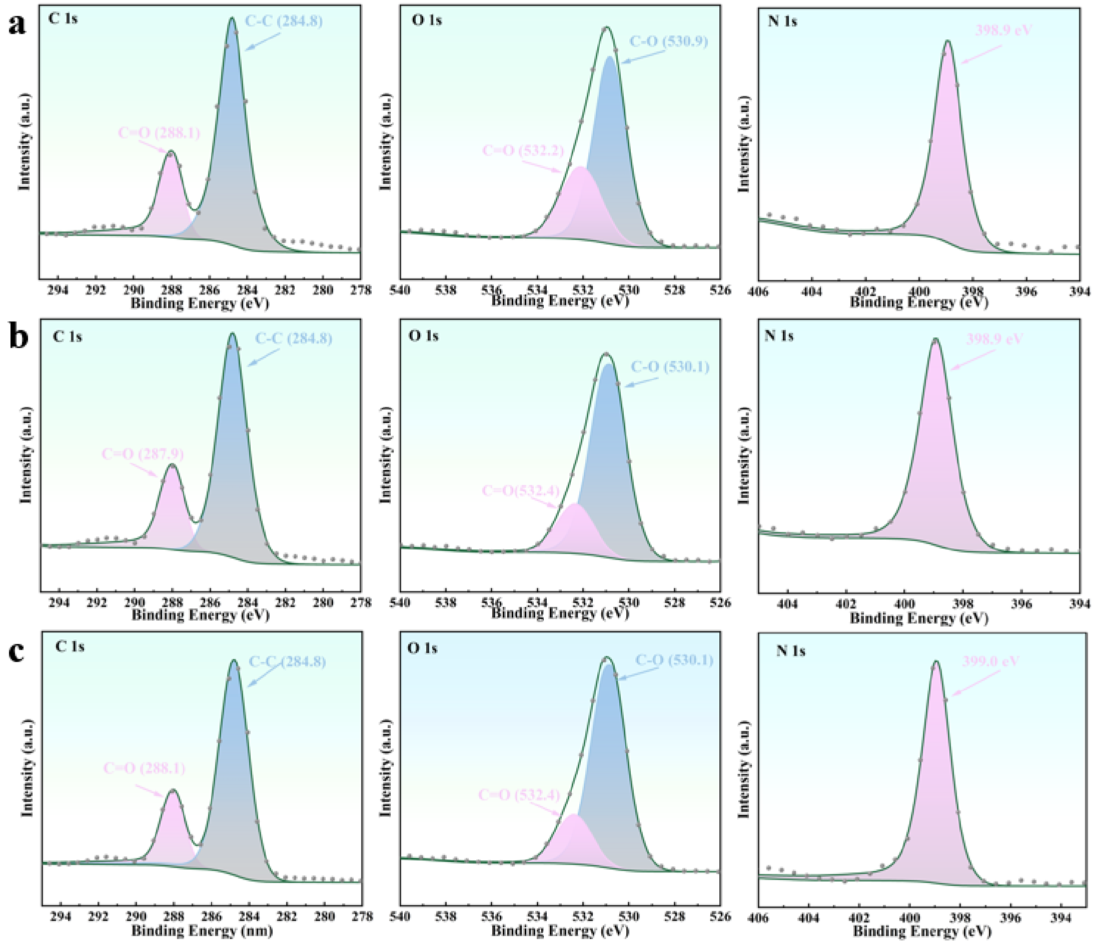


**Figure S2.** XPS C 1s, N 1s, and O 1s fitting results of 0/10 Eu/Tb (a); 5/5 Eu/Tb (b); 10/0 Eu/Tb (c).

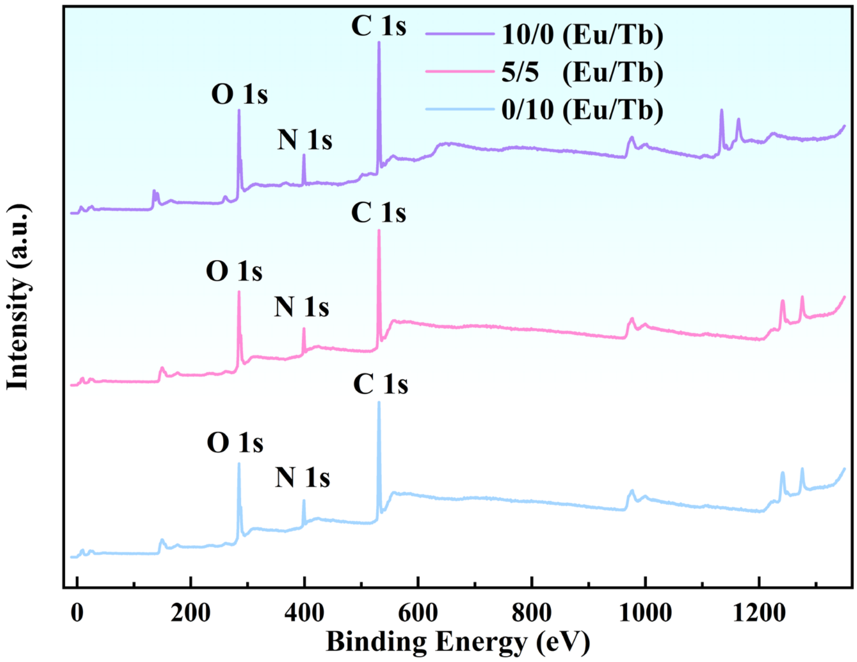


**Figure S3.** XPS scan of Ln-L3 (10/0 Eu/Tb; 5/5 Eu/Tb; 0/10 Eu/Tb).

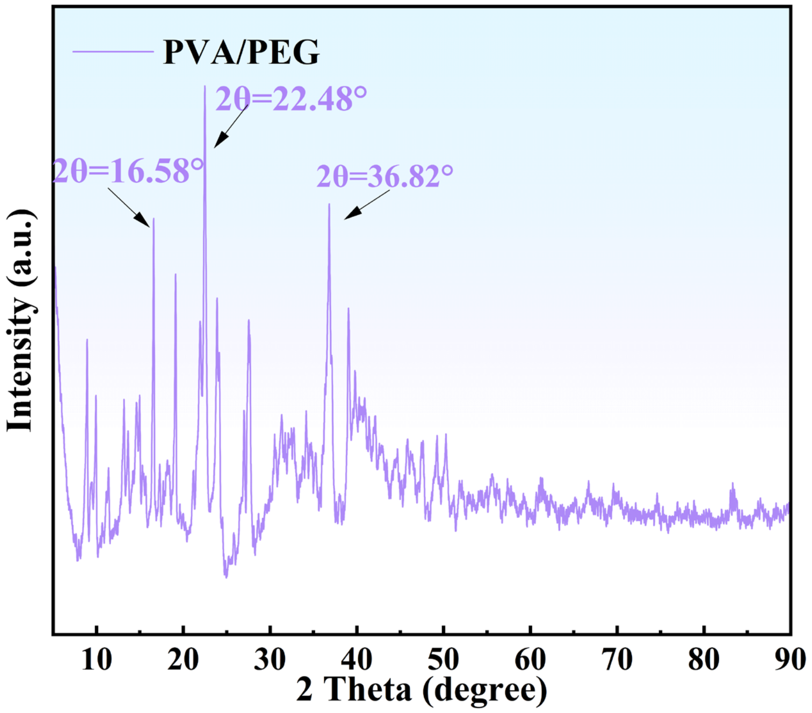


**Figure S4.** XRD patterns of PVA/PEG hydrogel.

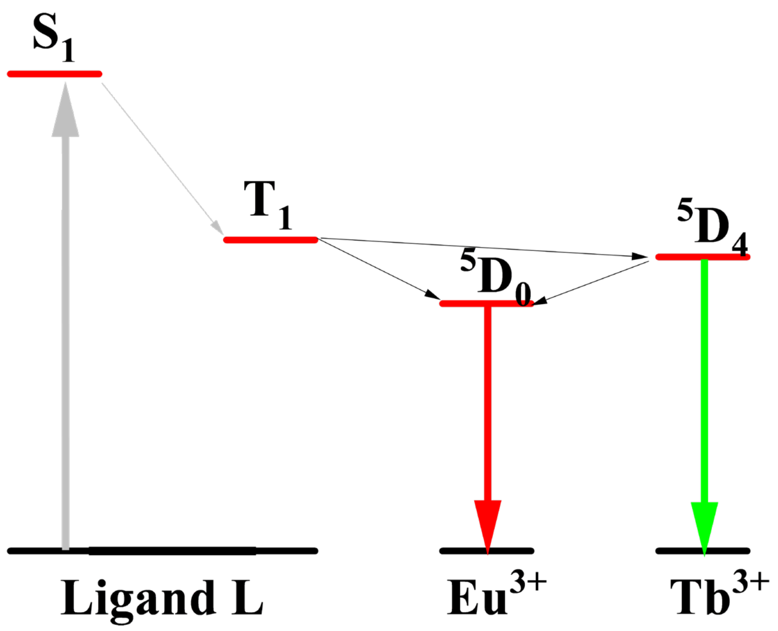


**Figure S5.** The energy transfer diagram.
